# 3'-Sialyllactose and *B. infantis* synergistically alleviate gut inflammation and barrier dysfunction by enriching cross-feeding bacteria for short-chain fatty acid biosynthesis

**DOI:** 10.1080/19490976.2025.2486512

**Published:** 2025-04-07

**Authors:** Mingzhi Yang, Zipeng Jiang, Lutong Zhou, Nana Chen, Huan He, Wentao Li, Zhixin Yu, Siming Jiao, Deguang Song, Yizhen Wang, Mingliang Jin, Zeqing Lu

**Affiliations:** a College of Animal Sciences, Zhejiang University, Hangzhou, China; b Key Laboratory of Molecular Animal Nutrition, Ministry of Education, Hangzhou, China; c Key Laboratory of Animal Nutrition and Feed Science (Eastern of China), Ministry of Agriculture and Rural Affairs, Hangzhou, China; d National Engineering Research Center of Green Feed and Healthy Breeding, Hangzhou, China; e Zhejiang Key Laboratory of Nutrition and Breeding for High-Quality Animal Products, Hangzhou, China; f Institute of Process Engineering, Chinese Academy of Sciences, Beijing, China; g Department of Immunobiology, Yale School of Medicine, New Haven, CT, USA

**Keywords:** 3'-Sialyllactose, *Bifidobacterium infantis*, ulcerative colitis, short-chain fatty acid, gut microbiota, synbiotic therapy, cross-feeding, intestinal barrier

## Abstract

Ulcerative colitis (UC) poses significant threats to human health and quality of life worldwide, as it is a chronic inflammatory bowel disease. 3'-sialyllactose (3'−SL) is a key functional component of milk oligosaccharides. This study systematically evaluates the prebiotic effects of 3'-SL and its therapeutic potential in combination with *Bifidobacterium infantis* (*B. infantis*) for UC. The findings reveal that 3'-SL and *B. infantis* synergistically mitigate intestinal inflammation and barrier dysfunction by promoting the production of short-chain fatty acids (SCFAs) through cross-feeding mechanisms among gut microbiota. Individually, 3'-SL, *B. infantis*, and the synbiotic treatment all effectively alleviated UC symptoms, including reduced weight loss, improved disease activity scores, and prevention of colon shortening. Histopathological and immunofluorescence analyses further demonstrated that the synbiotic treatment significantly ameliorated colonic injury, enhanced barrier function, restored goblet cell counts, increased glycoprotein content in crypt goblet cells, and upregulated the expression of tight junction proteins (ZO-1, occludin, and claudin-1). Notably, the synbiotic treatment outperformed the individual components by better restoring gut microbiota balance, elevating SCFA levels, and modulating serum cytokine profiles, thereby reducing inflammation. These findings provide mechanistic insights into the protective effects of the synbiotic and underscore its therapeutic potential for UC and other intestinal inflammatory disorders.

## GRAPHICAL ABSTRACT

**Figure:**
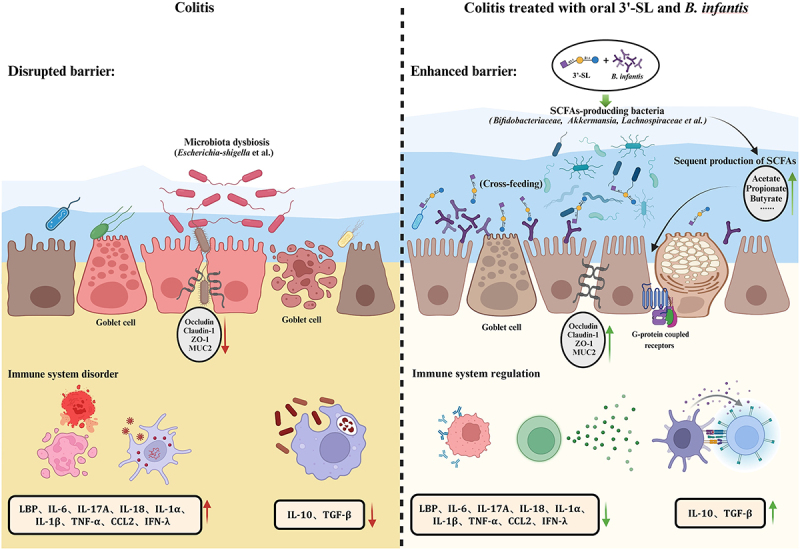

## Introduction

Inflammatory bowel disease (IBD), which includes ulcerative colitis (UC) and Crohn’s disease (CD), is a chronic inflammatory disorder of the gastrointestinal tract, affecting approximately 6 to 8 million individuals worldwide.^1–3^ UC, in particular, is characterized by periodic mucosal inflammation confined to the colon, with its incidence and prevalence steadily increasing worldwide.^4^ Despite extensive research, the precise etiology of UC remains unclear. Current evidence points to a multifaceted interplay of genetic predisposition, immune dysregulation, alterations in gut microbiota, and environmental triggers.^5–7^ Conventional treatments, such as aminosalicylates and immunomodulators, are often limited by suboptimal efficacy and adverse effects, necessitating the development of safer and more effective therapeutic strategies.^8^

Human milk oligosaccharides (HMOs), bioactive carbohydrates naturally found in breast milk, have emerged as promising candidates for gut health modulation due to their prebiotic properties and immunoregulatory functions. Among them, 3'-sialyllactose (3'−SL) is one of the most abundant and simplest sialylated HMOs, playing a critical role in shaping gut microbiota during infancy.^9,10^ Additionally, *Bifidobacterium longum* subspecies *infantis* (*B. infantis*), a predominant early gut colonizer in breastfed infants, efficiently metabolizes HMOs, contributing to gut epithelial barrier integrity and immune system development.^11^ The genome of *B. infantis* encodes specialized Gene clusters (H1 to H5) participating in HMO transport and intracellular metabolism, enabling the breakdown of complex carbohydrates via ABC transporters, major facilitator superfamily permeases, and phosphotransferase systems. These carbohydrates are further processed by glycosyl hydrolases into simpler forms, which subsequently enter energy production-associated catabolic pathways.^12–14^

The interaction between HMOs and *Bifidobacterium* species presents a promising avenue for novel therapies. HMOs not only act as prebiotics to promote the growth of commensal bacteria but also serve as decoy receptors, preventing pathogen adhesion to intestinal epithelial cells.^15–17^ Notably, 3'-SL has demonstrated immunomodulatory properties and the ability to selectively enhance the enrichment of *Bifidobacterium* species, which dominate the gut microbiota in breastfed infants and contribute to immune homeostasis.^10,18^ While both 3'-SL and *B. infantis* independently exhibit anti-inflammatory and microbiota-modulating effects, the mechanisms underlying their synergistic therapeutic potential in intestinal inflammation remain poorly understood, warranting further investigation.

Building on the established benefits of 3'-SL and *B. infantis*, we hypothesized that their combined administration would provide a synergistic therapeutic effect in UC management. This could be achieved by modulating the gut microbiota, reducing inflammation, and improving gut barrier function. Using a DSS-induced colitis mouse model, this study systematically evaluated the effects of 3'-SL and *B. infantis*, both individually and in combination. We determined the optimal dosages for each treatment and assessed their impact on colonic health through histopathology, immunofluorescence, 16S rRNA sequencing, short-chain fatty acid quantification, and serum cytokine analysis. This work aims to elucidate the therapeutic potential and the mechanisms underlying the combined use of 3'-SL and *B. infantis* in UC treatment.

## Materials and methods

### Characterization of 3'-sialyllactose

3'-Sialyllactose (3'−SL), with the structural formula Neu5Acα2,3 Galβ1,4Glc, is a sialylated human milk oligosaccharide characterized by an α2,3-linked glycosidic bond between N-acetyl-D-neuraminic acid (Neu5Ac or SA) and the galactose unit of lactose. This sialic acid-lactose conjugate is one of the most abundant HMOs (Supplementary Figure S1a). Scanning electron microscopy (SEM) imaging revealed the characteristic cloud-like structure of 3'-SL produced by the engineered bacterium (Supplementary Figure S1b), confirming the successful production and structural integrity of the compound. In this study, the 3'-SL sample was sourced from Glycom A/S, Denmark, and its purity was assessed using UPLC-MS/MS (Supplementary Method 1), revealing a purity greater than 95% (Supplementary Figure S1c). 3'-SL was biosynthesized using an engineered *Escherichia coli* K-12DH1 MDO strain (MAP425). This strain contained targeted insertions of truncated bacterial α-2,3-sialyltransferase genes and a bacterial-derived gene cluster encoding enzymes required for synthesizing activated sialic acid (α-N-acetylglucosamine-6-phosphate epimerase, sialic acid synthase, and CMP-Neu5Ac).

### Bacterial strains preparation

*Bifidobacterium longum subsp. infantis* EVC001 (*B. infantis*) used in this study was provided by Infinant Health, USA. This strain has been reported to possess the full genetic capability to degrade all glycosidic bonds of HMOs, as documented in previous studies.^19,20^ It is an anaerobic, Gram-positive bacterium characterized by its distinctive bifurcated morphology, typically appearing in “Y”- or “V”-shaped forms (Suppl. Figure S1d,e).

*B. infantis* was cultured in TPY (Tryptone-Yeast Extract) liquid medium at 37°C under anaerobic conditions in a workstation (ELECTROTEK AW200SG, England). After activation, a 1% inoculum of the activated culture was transferred into 200 mL of TPY medium. Growth was monitored by measuring the optical density at 600 nm (OD600) every 2 h over a 24-h period. *B. infantis* reached a growth plateau at 16 h in TPY medium (Suppl. Figure S1f).

The *B. infantis* strain exhibited robust acid production, high bile salt tolerance, resistance to trypsin, and efficient HMO utilization (Suppl. Methods 2 to 4, Supplementary Figure S1g to S1j). In vitro experiments demonstrated that *B. infantis* could utilize 2‘-FL, 3'-FL, 3'-SL, 6‘-SL, LNT, and LNnT as sole carbohydrate sources in a modified MRS medium without added carbohydrates (Supplementary Table S1).

### Animal experiment design

A preliminary experiment was conducted to determine the optimal dosage for 3'-SL and *B. infantis* interventions (Suppl. Figure S2a). Specific pathogen-free (SPF) male C57BL/6J mice (6–7 weeks old, GemPharmatech Co., Ltd., Beijing, China) were housed under standard SPF conditions at Zhejiang University, China (12-h light/dark cycle, 22°C, and 50% humidity). Mice were randomly divided into eight groups (*n* = 8/group, 4 per cage). All mice were acclimatized for 3 days with unrestricted access to regular food and water. All animal treatments and experiments were conducted in accordance with the National Research Council’s *Guide for the Care and Use of Laboratory Animals* and approved by the Zhejiang University Scientific Ethics and Safety Committee (No. 2020-ZJU-17256; Hangzhou, China).

For the 3'-SL groups, 3'-SL was dissolved in 200 µL phosphate-buffered saline (PBS) at concentrations of 12.5 mg, 25 mg, and 50 mg. The solutions were administered daily via gavage for 3 weeks. For the probiotic group, *B. infantis* strains were grown separately and harvested during the late exponential phase. The cultures were washed with reduced PBS under anaerobic conditions, pelleted by centrifugation (8000 × g for 10 min), and then re-suspended in pre-reduced PBS to prepare bacterial suspensions with final concentrations of 1 × 10^8^, 1 × 10^9^, and 1 × 10^10^ colony-forming units (CFU) per 200 µL. These suspensions were administered via gavage daily for 3 weeks. Meanwhile, the control and model groups received 200 µL PBS daily for 3 weeks. To induce colitis, all groups except the control group were provided with drinking water containing 3% (w/v) dextran sulfate sodium (DSS, molecular weight 36–40 kDa; MP Biomedicals, Solon, OH, USA) during the final week of the experiment.

A functional experiment was conducted to explore the synergistic effects of 3'-SL and *B. infantis* on colitis. (Figure 2(a)). SPF male C57BL/6J mice (6–7 weeks old, *n* = 6/group) were used. For the 3'-SL group, 25 mg of 3'-SL was dissolved in 200 µL PBS and administered daily via gavage for 3 weeks. For the *B. infantis* group, 1 × 10^10^ CFU *B. infantis* suspensions were dissolved in 200 µL PBS and administered via gavage daily for 3 weeks. For the synbiotic group, 25 mg of 3'-SL and 1 × 10^10^ CFU of *B. infantis* were both dissolved in 200 µL PBS, and then administered via gavage daily for 3 weeks. Control and model groups received 200 µL PBS daily for 3 weeks. Colitis was induced in all groups except the control group by providing 3% DSS in drinking water during the final week of the experiment.

### Clinical and histologic parameter analysis

Body weight, stool consistency, fecal blood presence, and colon length were recorded by a blinded observer. The spleen index was calculated as the ratio of spleen weight to terminal body weight. The disease activity index (DAI) was determined by assessing weight loss, stool consistency, and fecal bleeding, as follows: Weight loss: 0 = none, 1 = 5–10%, 2 = 10–15%, 3 = 15–20%, 4 = >20%; Stool consistency: 0 = normal, 2 = sparse, 4 = diarrhea; Bleeding: 0 = none, 2 = blood present, 4 = gross blood. The DAI score was calculated as the sum of the three parameters, as described previously.^21^

Colon tissues were harvested, fixed in 4% paraformaldehyde for 72 h, and processed following standard paraffin-embedding protocols. Sections (5 μm thick) were stained with hematoxylin and eosin (H&E) and periodic acid-Schiff (PAS) to assess tissue morphology and goblet cell integrity. All stained slides were examined under a Nikon ECLIPSE E100 light microscope by blinded observers. Inflammatory infiltration, changes in crypt structure, ulceration, crypt loss, and edema were evaluated.

Histological scoring was conducted according to the criteria previously described.^22^ Three parameters were evaluated as follows: Inflammation severity: 0 = none, 1 = mild, 2 = moderate, 3 = severe; Extent of inflammatory involvement: 0 = none, 1 = mucosal, 2 = mucosal and submucosal, 3 = transmural; Degree of epithelial/Crypt damage: 0 = none, 1 = basal third, 2 = basal two-thirds, 3 = crypt loss, 4 = crypt and surface epithelial destruction. The scores for each parameter were summed to determine the overall histological score. The images were evaluated using the Image J software.

### Indirect immunofluorescence assay

Immunofluorescence was performed as previously described.^23^ The primary antibodies used were ZO-1 (Abcam, Catalog No. 307799), Occludin (Abcam, Catalog No. 216327), and Claudin-1 (Abcam, Catalog No. 307692). Fluorescently labeled intestinal proteins were visualized and digital images were captured using a fluorescence microscope (Nikon Eclipse C1).

### Measurement of occludin, claudin-1, and GAPDH expression in colon tissues by western blotting

Mouse colon tissue protein was extracted following the method described by previous studies.^23^ The primary antibodies used were Occludin (Abcam, Catalog No. 216327), Claudin-1 (Abcam, Catalog No. 307692), and GAPDH Rabbit pAb (Abclone, Catalog No. A19056). Immunoreactive bands were detected using an ECL system (ChemiScope 6100, Shanghai, China). The intensity of protein bands and fluorescence from independent Western blot (WB) experiments was quantified using ImageJ software.

### Ultrastructural examination of colon tissues, *B.* *infantis*, and 3'-SL

Colon tissues were prepared for transmission electron microscopy (TEM) using glutaraldehyde and osmium tetroxide fixation, ethanol gradient dehydration, resin infiltration, embedding, ultrathin sectioning, and staining with uranyl acetate and lead citrate, followed by imaging with a Hitachi HT7800 TEM.

SEM imaging of probiotics was performed after glutaraldehyde fixation, ethanol dehydration, critical point drying, and conductive coating (gold or platinum) to visualize bacterial surface morphology under low accelerating voltage. For 3'-SL samples, the preparation involved dissolving the oligosaccharide in distilled water, coating it onto a substrate, vacuum drying, applying a conductive coating (gold, platinum, or carbon), and imaging under optimized low-voltage SEM settings to analyze particle morphology and surface texture.

### Microbial DNA extraction, amplification, and analysis.

Microbial DNA was extracted from samples using the PowerSoil DNA Isolation kit (MoBio Laboratories, Carlsbad, CA, USA) following the manufacturer’s instructions, as described in previous studies.^23^ DNA concentration was measured with a NanoDrop 2000 spectrophotometer (Thermo Scientific, Wilmington, USA).

The V3-V4 region of the bacterial 16S rDNA gene was amplified using the universal primer set 338 F (5’-ACTCCTACGGGAGGCAGCA-3') and 806 R (5’-GGACTACHVGGGTWTCTAAT-3'). Sequencing was conducted on an Illumina NovaSeq 6000 platform (2 × 250 bp) at Beijing Biomarker Technologies Co., Ltd., Beijing, China. Raw sequencing data were processed using QIIME2, with the Cutadapt plugin for primer trimming and the DADA2 plugin for quality control and chimera removal. Sequences were clustered into Amplicon Sequence Variants (ASVs), and taxonomic classification was performed using Naive Bayes classifiers trained on the Ribosomal Database Project (v.11.5).

Denoising and ASV generation were performed using the DADA2 method in QIIME2 2020.6. Taxonomic annotation was carried out using Naive Bayes classifiers, with UNITE as the reference database. Gut microbiota diversity and abundance were assessed using alpha diversity metrics (Chao1 and Shannon index), and beta diversity was evaluated via Principal Coordinates Analysis (PCoA) based on Bray-Curtis distances or binary Jaccard. Permutational multivariate analysis of variance (PERMANOVA) was used to further analyze whether there were significant differences in sample composition between different treatment groups. Linear discriminant analysis (LDA) and LDA effect size (LEfSe) were applied to identify metagenomic biomarkers between groups, with an LDA score threshold of 4.0. Some data analysis was conducted on the BioMarker online cloud platform (https://www.biocloud.net/), provided by BioMarker Technologies (Beijing, China).

### Serum cytokine quantification by ELISA

Whole blood was collected from each mouse and centrifuged at 2500 ×g for 15 min at 4°C to obtain approximately 200 μL of serum. Levels of pro-inflammatory cytokines (IL-6, IL-17A, IL-18, IL-1α, IL-1β, TNF-α, CCL2), anti-inflammatory cytokines (IL-10, TGF-β), and immunoregulatory factors (IFN-λ) in the serum were measured using ELISA kits (Jiangsu Jingmei Biotechnology Co., Ltd., China) according to the manufacturer’s instructions. After stopping the color development reaction, absorbance was measured at 450 nm using a microplate reader (BioTek Synergy HTX, Winooski, VT, USA).

### Quantitative real-time PCR

Total RNA from the colon was extracted using the SteadyPure RNA Extraction Kit (AG, Hunan). The quantity of the extracted RNA was assessed using the NanoDrop 2000 (Thermo Fisher Scientific). Subsequently, 1 µg of RNA was reverse transcribed using a reverse transcription kit (Takara, Kusatsu, Japan). Quantitative real-time PCR (qRT-PCR) was performed with gene-specific primers (Suppl. Table S2) and SYBR Green dye (Takara, Kusatsu, Japanese) using a StepOnePlus Real-Time PCR system (Applied Biosystems, Foster City, USA). The procedure involved an initial denaturation at 95°C for 30 s, followed by 40 cycles of denaturation at 95°C for 5 s, annealing at 60°C for 30 s, and extension at 60°C for 30 s. The relative mRNA expression of the target gene was analyzed using the 2^−∆∆CT^ method, with GAPDH serving as the housekeeping gene.

### Quantification of SCFAs profiles

Fecal samples were collected separately from each mouse using a sterile, freeze-storage tube. Following a previously reported method,^24^ 0.1 g cecal contents sample from each mouse was initially mixed thoroughly with 1 mL of methanol and allowed to stand for 5–10 min. Subsequently, the pH value of the mixture was adjusted to 2–3 with 1% sulfuric acid, and then shaken to ensure thorough mixing before standing for an additional 5–10 min. The mixture was then centrifuged at 5,000 × g for 20 min at 4°C, and the supernatant was transferred to a new microtube for SCFAs detection. GC-MS analysis was conducted using a 7890B gas chromatograph coupled with a 5977 single quadrupole mass spectrometer (Agilent Technologies, Santa Clara, CA, USA) with a DB-FFAP125-3237 capillary column (Agilent Technologies). Specifically, the column temperature was set to 200°C, pressure at 100 kPa, and total flow at 63 mL/min. Data were analyzed using Agilent Mass Hunter software and the SCFAs content was calculated using external standard methods.

### Statistical analysis

Statistical analyses were conducted using GraphPad Prism 6.0 (GraphPad Software, San Diego, CA, USA). Data are presented as mean ± standard deviation (SD). Student’s t-test was used to compare two groups, while one-way ANOVA with post hoc comparisons was applied for multiple groups. Additionally, two-way ANOVA was employed to assess the interaction effects between 3'-SL and *B. infantis*. Statistical significance was considered at *p* < 0.05, with the following significance levels: *p < 0.05, **p < 0.01, ***p < 0.001, ****p < 0.0001, and “ns” indicating no significant difference. All experiments were repeated independently at least three times to ensure reproducibility.

## Results

### Study on the optimal dose of 3'-SL and *B.* *infantis* for colitis treatment.

To identify the optimal doses of 3'-SL and *B. infantis* for DSS-induced colitis treatment (Supplementary Figure S2a), we tested various doses and observed their effects on body weight, colon damage, liver and spleen condition, and disease indices in mice. Suppl. Figure S2b shows that daily administration of 3'-SL (25 mg/day) and *B. infantis* (1 × 10^10^ CFU/day) significantly reduced weight loss, with the most pronounced effects seen at these doses.

Anatomical analysis revealed that 3'-SL (25 mg/day), *B. infantis* (1 × 10^9^ and 1 × 10^10^ CFU/day) significantly reduced colon hemorrhage, edema, and shortening. The other doses, including 3'-SL (12.5 mg/day, 50 mg/day) and *B. infantis* (1 × 10^8^ CFU/day), did not significantly affect these parameters (Supplementary Figure S2c).

Compared to the control group, the model group exhibited splenomegaly and reduced liver weight. Administration of *B. infantis* (1 × 10^10^ CFU/day) effectively mitigated both splenomegaly and liver damage (Suppl. Figures S2d and S2e). Additionally, 3'-SL (25 mg/day) helped alleviate liver weight loss, while other doses showed no such effects.

DSS induction increased disease activity index (DAI) and histological scores (Suppl. Figures S2f and S2g). A low dose of 3'-SL (12.5 mg/day) reduced the DAI but had no significant effect on histology. Conversely, *B. infantis* (1 × 10^8^ CFU/day) improved histology without changing DAI. The greatest improvements in DAI and histology scores were seen with 3'-SL (25 mg/day) and *B. infantis* (1 × 10^10^ CFU/day). H&E and PAS staining (Suppl. Figure S2h) showed that 3'-SL (25 mg/day) and *B. infantis* (1 × 10^10^ CFU/day) were the most effective, reducing inflammatory cell infiltration, submucosal granulomas, and restoring mucosal integrity.

Overall, the results indicate that different doses of 3'-SL or *B. infantis* exhibited varying therapeutic effects against DSS-induced colitis in mice. The most significant benefits were observed with 3'-SL (25 mg/day) and *B. infantis* (1 × 10^10^ CFU/day). These optimal doses effectively alleviated weight loss, reduced colon damage, mitigated splenomegaly and liver weight reduction, and improved both histological and disease activity index scores. Furthermore, their ability to reduce inflammatory cell infiltration, alleviate submucosal granulomas, and restore intestinal structure underscores their potential as promising interventions for colitis treatment.

### Effect of 3'-SL, *B.* *infantis*, and the synbiotic on DSS-induced colitis in mice.

**Figure 1.**
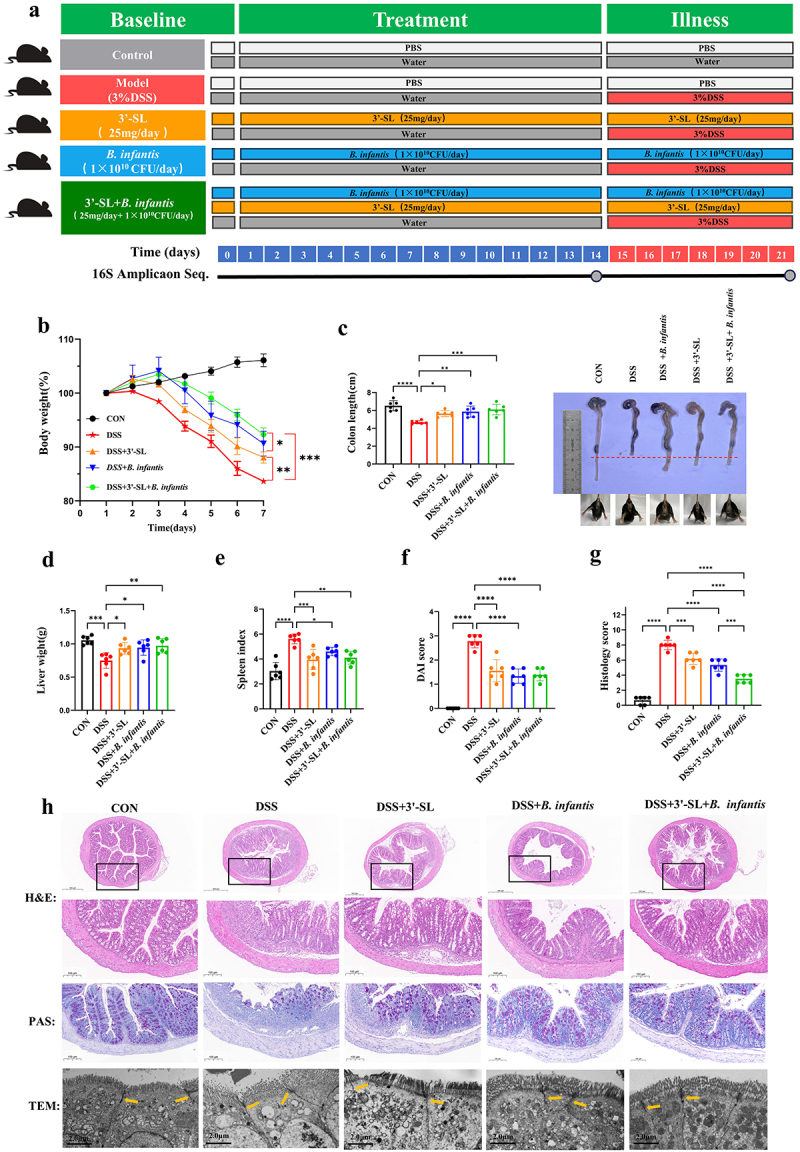
3'-SL, *B. infantis*, and the synbiotic variably mitigated DSS-induced colitis symptoms (*n* = 6 per group). (a) Experimental design. (b) Body weight changes of mice after receiving different treatments and induction of colitis. (c) Colon length in each group and representative images of mouse colons. (d) Liver weight in different mouse groups. (e) Spleen index in different mouse groups. (f) DAI scores in different mouse groups on the final experimental day. (g) Histology scores in different mouse groups. (h) H&E and PAS staining, along with TEM analysis of colon sections in different mouse groups. Yellow markers indicate tight junction structures in the colon under TEM. Scale bars: 500 μm (upper panel), 100 μm (middle panel), and 2 μm (lower panel).

To evaluate whether the synbiotic treatment could achieve superior therapeutic efficacy, an experiment was conducted (Figure 1(a)), building on previous dose-detection studies. During the experiment, no significant differences in water intake were observed, confirming that all groups received the same DSS dose.

Compared to 3'-SL or *B. infantis* alone, the synbiotic treatment was the most effective in alleviating DSS-induced weight loss in mice (Figure 1(b)). Anatomical observations revealed that 3'-SL, *B. infantis*, and the synbiotic treatment all significantly alleviated DSS-induced colonic hemorrhage and shortening, reduced liver weight loss, suppressed splenomegaly, and decreased DAI scores, indicating an overall improvement in the inflammatory response and disease severity (Figures 1(c–f)). However, when compared to 3'-SL or *B. infantis* alone, the synbiotic treatment did not result in significant changes in these parameters.

Histopathological analysis further confirmed that synbiotic treatment offers a better protective effect against DSS-induced colitis. H&E and PAS staining revealed normal colon morphology in the control group, characterized by intact mucosa and orderly crypt architecture. In contrast, the model group exhibited epithelial erosion, crypt destruction, muscularis mucosae damage, submucosal edema, and inflammatory cell infiltration (Figures 1(g,h)). Treatment with 3'-SL, *B. infantis*, or the synbiotic ameliorated these pathological changes, with the synbiotic showing the greatest efficacy. Synbiotic-treated mice exhibited significantly reduced inflammatory cell infiltration, decreased submucosal granulomas, reduced intestinal wall thickening, and improved cytoplasmic glycogen levels.

Transmission electron microscopy analysis revealed that DSS treatment caused shortening of intestinal epithelial villi, disruption of the brush border, and damage to tight junctions (Figure 1(h)). Treatment with 3'-SL or *B. infantis* preserved the tight junctions between adjacent cells and effectively restored the length, density, and arrangement of colon microvilli in mice, with the synbiotic demonstrating the most substantial improvement.

Collectively, all treatments significantly alleviated DSS-induced colonic hemorrhage, shortening, liver weight loss, splenomegaly, and reduced DAI scores. However, the synbiotic treatment proved to be more effective than 3'-SL or *B. infantis* alone in alleviating DSS-induced weight loss in mice. The synbiotic also showed the greatest improvement in tissue integrity by reducing inflammatory cell infiltration, submucosal granulomas, and intestinal wall thickening. TEM analysis further revealed that the synbiotic most effectively restored microvilli structure and tight junctions. These results highlight the enhanced therapeutic benefits of the synbiotic, particularly in mitigating DSS-induced weight loss and promoting intestinal repair.

#### Effects of 3'-SL, *B.* *infantis*, and the synbiotic on intestinal barrier function.

**Figure 2.**
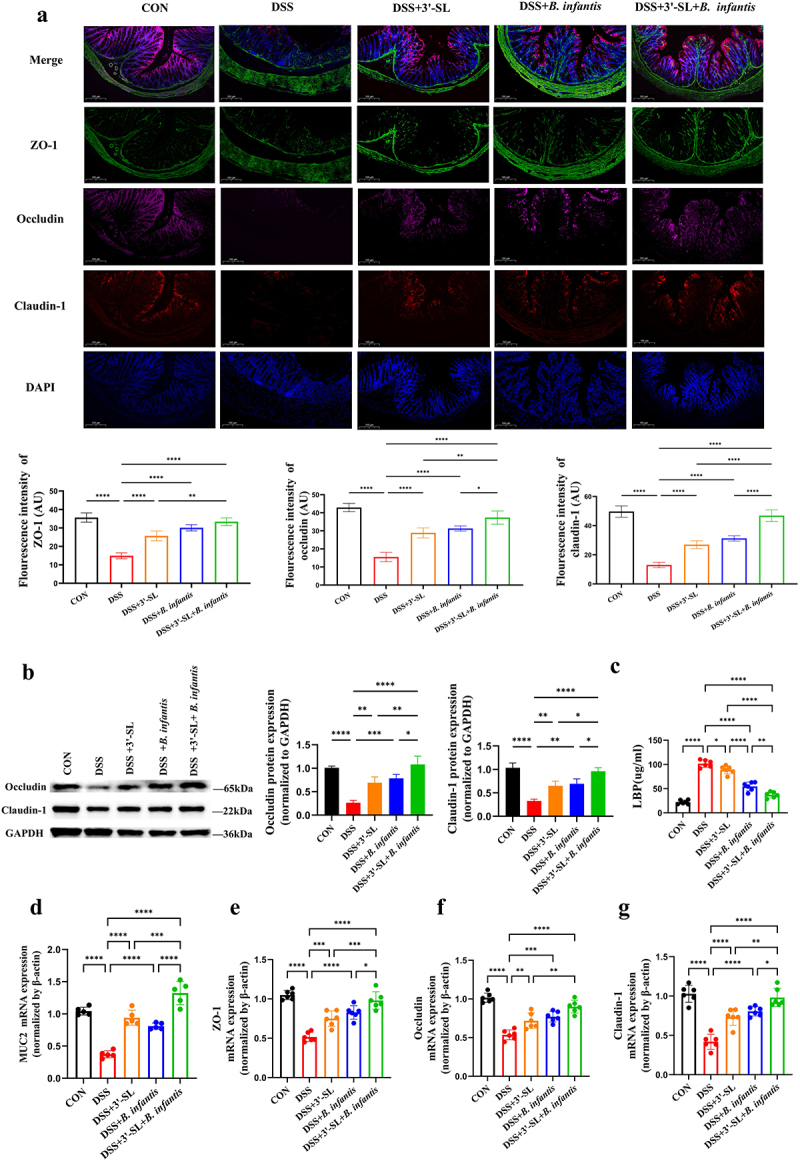
3 ’-SL, *B. infantis*, and the synbiotic differentially affect gut barrier function (*n* = 6 per group). (a) Immunofluorescence analysis of tight junction protein expression in colon tissues from mice. Claudin-1 (red), Occludin (pink), ZO-1 (green), and DAPI (blue). Scale bar = 100 μm. (b) Expression levels of tight junction proteins claudin-1 and Occludin in colon tissues of mice. (c) Serum levels of LPS-binding protein (LBP) as determined by ELISA. (d) mRNA expression levels of MUC2 as measured by qRT-PCR. (e) mRNA expression levels of ZO-1 as measured by qRT-PCR. (f) mRNA expression levels of Occludin as measured by qRT-PCR. (g) mRNA expression levels of Claudin-1 as measured by qRT-PCR.

Immunofluorescence analysis confirmed that DSS reduced the fluorescence intensity of occludin, claudin-1, and ZO-1, which was significantly restored by synbiotic treatment. The effects of 3'-SL and *B. infantis* were comparable, but less pronounced than those of the synbiotic (Figure 2(a)). Similarly, western blot analysis showed that DSS treatment downregulated occludin and claudin-1 protein expression in colonic tissues. Treatment with 3'-SL, *B. infantis*, and the synbiotic upregulated these proteins, with the synbiotic group exhibiting the most pronounced effect (Figure 2(b)).

In addition, impaired intestinal barrier function was assessed by measuring plasma LPS-binding protein (LBP) levels. Compared with the control group, plasma LBP levels were significantly elevated in the DSS-induced group (*p* < 0.0001, CON vs. DSS). Treatment with 3'-SL, *B. infantis*, and the synbiotic significantly reduced LBP levels, with the greatest reduction observed in the synbiotic group (Figure 2(c)).

To further assess the effects on gut barrier integrity, the mRNA expression levels of ZO-1, occludin, claudin-1, and Mucin 2 were analyzed. Compared to the control group, DSS treatment resulted in significant reductions in the mRNA expression of ZO-1, occludin, claudin-1, and Mucin 2 by 48.6%, 49.0%, 59.8%, and 98.5%, respectively (Figures 2(d–g)). Treatment with 3'-SL, *B. infantis*, and the synbiotic significantly increased the expression of these genes, with the synbiotic showing the most pronounced enhancement.

Collectively, these findings suggest that both 3'-SL and *B. infantis* can enhance the expression of tight junction proteins and improve gut barrier integrity in DSS-induced colitis. Compared to treatments with either 3'-SL or *B. infantis* alone, the synbiotic formulation exhibited superior therapeutic efficacy in maintaining colon barrier function.

#### Effects of 3'-SL, *B.* *infantis*, and the synbiotic on serum inflammatory cytokines.

**Figure 3.**
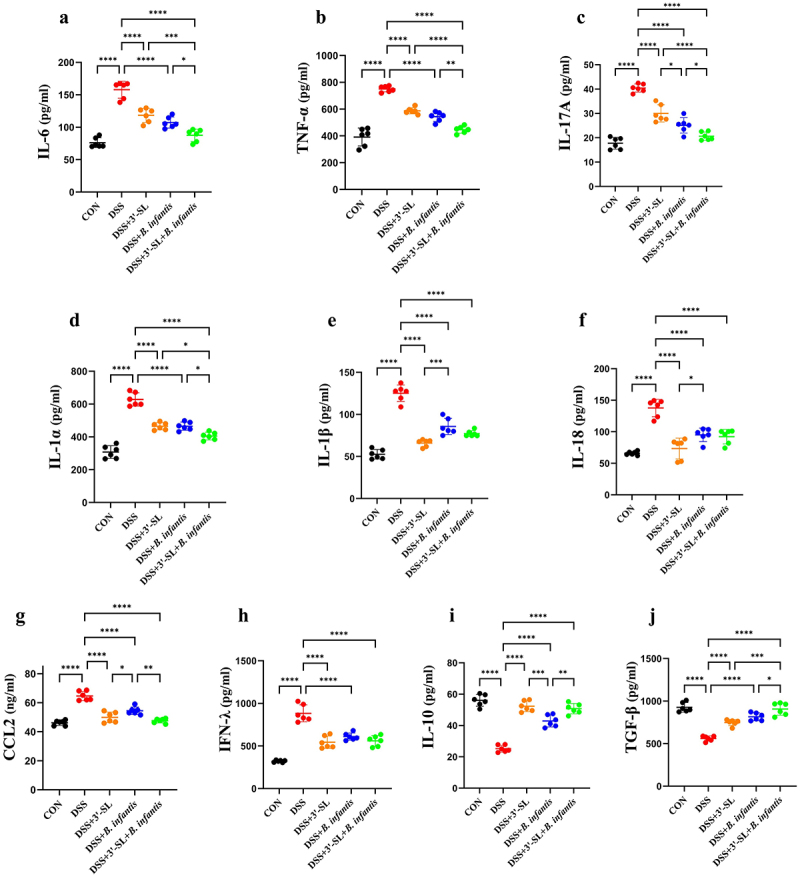
Treatment with 3'-SL, *B. infantis*, and the synbiotic differentially affects the expression of serum cytokines (*n*=6 per group). Serum concentrations of (a) IL-6, (b) TNF-α, (c) IL-17A, (d) IL-1α, (e) IL-1β, (f) IL-18, (g) CCL2, (h) IFN-λ, (i) IL-10, and (j) TGF-β in mice treated with 3'-SL, *B. infantis*, and the synbiotic.

To evaluate the effects of 3'-SL, *B. infantis*, and the synbiotic on the inflammatory response, serum levels of inflammatory cytokines were measured in mice. As shown in Figure 3, DSS treatment significantly upregulated the pro-inflammatory cytokines IL-6, IL-17A, IL-1α, IL-1β, IL-18, TNF-α, and CCL2, as well as the immunoregulatory factor IFN-λ (Figures 3(a–h)), while significantly downregulating the anti-inflammatory factors IL-10 and TGF-β (Figures 3(i,j)).

Treatment with 3'-SL, *B. infantis*, and the synbiotic formulation effectively mitigated the DSS-induced changes in cytokine levels, although the effectiveness of each treatment varied.

Compared to *B. infantis* treatment, 3'-SL was more effective in preventing the upregulation of IL-1β, IL-18, and CCL2 induced by DSS, as well as effectively preventing the downregulation of IL-10. In contrast, compared to the 3'-SL group, *B. infantis* treatment better prevented the upregulation of IL-17A induced by DSS, suggesting distinct mechanisms in regulating the inflammatory balance.

Notably, compared to treatments with 3'-SL or *B. infantis* alone, the synbiotic formulation significantly inhibited the DSS-induced upregulation of serum IL-6, TNF-α, IL-17A, IL-1α, and CCL2, while most effectively restoring the levels of TGF-β and IL-10, demonstrating the strongest overall anti-inflammatory effect.

In conclusion, both 3'-SL and *B. infantis* demonstrated significant, albeit distinct, effects in modulating the inflammatory response in the context of DSS-induced colitis. 3'-SL was particularly effective in mitigating the DSS-induced upregulation of IL-1β, IL-18, and CCL2, while *B. infantis* more efficiently prevented the DSS-induced upregulation of IL-17A, suggesting differential mechanisms in regulating the inflammatory cascade. Notably, the synbiotic treatment exhibited the most robust anti-inflammatory effects by significantly inhibiting the DSS-induced upregulation of several pro-inflammatory cytokines (including IL-6, TNF-α, IL-17A, IL-1α, and CCL2) and effectively restoring the levels of anti-inflammatory cytokines such as IL-10 and TGF-β, highlighting its potential as a more effective therapeutic strategy for managing gastrointestinal inflammation.

#### Effect of 3'-SL, *B.* *infantis*, and the synbiotic on SCFA levels and GPCR expression.

**Figure 4.**
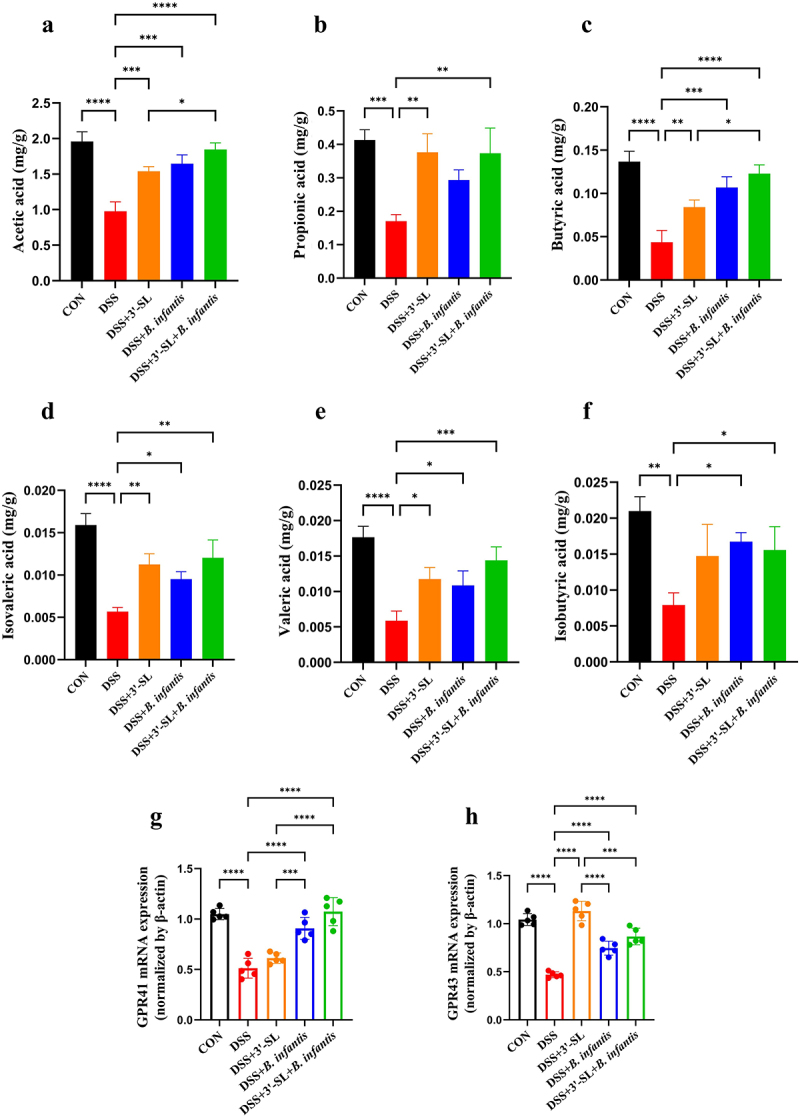
Effects of 3'-SL, *B. infantis*, and the synbiotic treatments on SCFA production and GPCR expression in the cecum and colon tissues (*n* = 5 per group). (a) Levels of acetic acid. (b) Levels of propionic acid. (c) Levels of butyric acid. (d) Levels of valeric acid. (e) Levels of isovaleric acid. (f) Levels of isobutyric acid. (g) Expression of the G-protein-coupled receptors GPR41 in colon tissues, as determined by qRT-PCR. (h) Expression of the G-protein-coupled receptors GPR43 in colon tissues, as determined by qRT-PCR.

To investigate the correlation between gut microbiota and colon health, SCFA levels in the cecum were analyzed. As shown in Figure 4(a), acetic acid was the most abundant SCFA in the cecum of mice, as expected. The levels of acetic acid, propionic acid, butyric acid, isobutyric acid, valeric acid, and isovaleric acid in the DSS group were significantly lower than those in the CON group (Figures 4(a–f)).

Compared to the control group, treatment with either 3'-SL or *B. infantis* alone effectively increased the production of various SCFAs in the cecum, with no significant difference observed between the two. Notably, the synbiotic treatment resulted in the most pronounced increase in both acetic acid and butyric acid production, surpassing the effects of either 3'-SL or *B. infantis* alone.

Given that SCFAs are recognized by G-protein coupled receptors (GPCRs), including GPR41 and GPR43, their expression in colon tissues was examined via qRT-PCR. As shown in Figures 4(g,h) the expression of GPR41 and GPR43 was significantly reduced in the model group compared to the control group at the mRNA level. 3'-SL treatment significantly increased GPR43 expression but did not affect GPR41. In contrast, both *B. infantis* and the synbiotic significantly increased GPR41 expression, with the synbiotic showing the most substantial effect (Figures 4(g,h)).

These findings suggest that 3'-SL primarily exerts its anti-inflammatory effects through GPR43 activation, whereas *B. infantis* and the synbiotic formulation activate GPR41 in colonic cells. These effects are likely mediated by changes in gut microbiota composition and enhanced SCFA production. Notably, the synbiotic formulation, by synergistically combining the distinct effects of 3'-SL and *B. infantis*, enhances the production of key SCFAs like acetic acid and butyric acid, leading to a more robust modulation of inflammatory pathways. This dual approach offers a more effective strategy for restoring gut homeostasis and improving colon health compared to single treatments.

#### Effects of 3'-SL, *B.* *infantis*, and the synbiotic on gut microbiota in normal mice.

To evaluate the impact of 3'-SL, *B. infantis*, and the synbiotic on gut microbiota composition in normal mice, we collected mouse feces before the challenge for 16S rRNA gene amplicon sequencing. α-Diversity, assessed using the Chao1 and Shannon indices, represents microbial richness and diversity at the genus level and was analyzed using the Wilcoxon rank-sum test.

**Figure 5.**
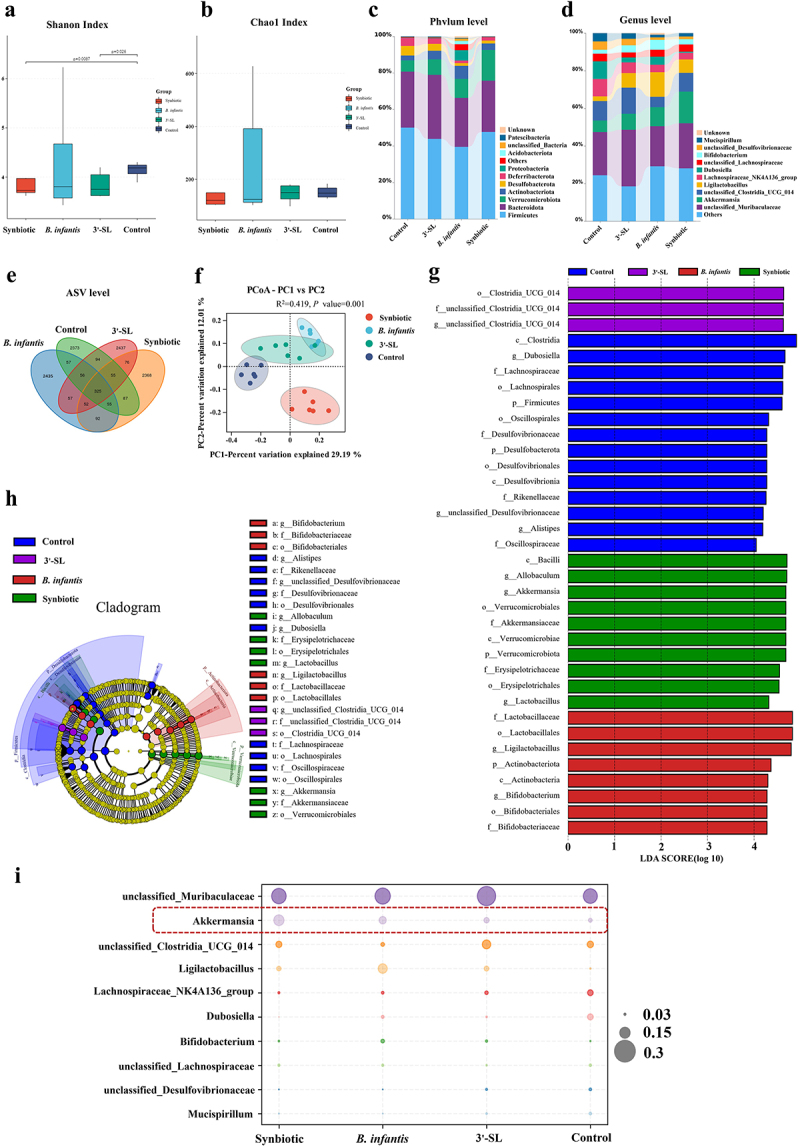
The composition of gut microbiota in normal mice administered 3'-SL, *B. infantis*, and the symbiotic (*n* = 6 per group). Mice were administered PBS, 3'-SL, *B. infantis*, and the synbiotic for 14 days, and fecal samples were collected for 16s rRNA sequencing. Alpha-diversity is represented by the (a) Chao1 index and (b) Shannon index. (c) Phylum level. (d) Taxonomic level. (e) Venn diagram showing the common and unique amplicon sequence variants (ASVs) in the four groups. (f) PCoA plots based on Bray-Curtis distance of the four groups. (g) LEfSe plots of different microbiota based on ASV level (LDA > 4) of the four groups. (h) Evolutionary branch diagram of LefSe analysis based on classification information. (i) Fecal microbiota abundance at the genus level among all groups. Additional note: some taxa, such as unclassified Clostridia UCG-014 and unclassified Muribaculaceae, were classified at the family level but are displayed in the genus-level bar plots due to database limitations. These taxa were included in the visualization to maintain consistency across all taxonomic levels.

As shown in Figures 5(a,b) the synbiotic or 3'-SL intervention significantly reduced the Shannon index (3'-SL vs. CON, *p* = 0.026; synbiotic vs. CON, *p* = 0.0087) but did not significantly affect the Chao1 index. In contrast, *B. infantis* intervention did not significantly affect either the Shannon or the Chao1 index.

At the phylum level (Figure 5(c)), Firmicutes, Bacteroidota, Verrucomicrobiota, Actinobacteriota, Desulfobacterota, and Deferribacterota were the predominant phyla identified across all samples. The combination of 3'-SL and *B. infantis* reduced the relative abundances of Firmicutes and Bacteroidota, which was associated with an increase in Actinobacteriota and Verrucomicrobiota relative to the control group.

At the taxonomic level analyzed (Figure 5(d)), significant variations were detected among the treatment groups. 3'-SL treatment increased the relative abundance of several beneficial taxa, including unclassified Muribaculaceae, unclassified Clostridia_UCG 014, *Akkermansia*, *Ligilactobacillus*, and *Bifidobacterium*. Similarly, the *B. infantis* group exhibited a significant increase in the relative abundance of *Akkermansia*, *Ligilactobacillus*, and *Bifidobacterium*. Notably, while the abundance of *Bifidobacterium* also increased in the synbiotic group, this group exhibited the highest relative abundance of *Akkermansia* at 16.7%, compared to 8.5% in the *B. infantis* group and 11.7% in the 3'−SL group.

Venn diagram analysis indicated that the synbiotic group had a similar ASV number to the control group (2368 vs. 2373), while the 3'-SL and *B. infantis* groups exhibited comparable ASV numbers (2437 vs. 2435) (Figure 5(e)).

Principal Coordinate Analysis (PCoA) based on Bray–Curtis distance revealed distinct clustering of gut microbiota in the synbiotic group compared to the CON, 3'-SL, and *B. infantis* groups, indicating significant alterations in the microbiota structure at the ASV level (Figure 5(f)). PERMANOVA analysis further confirmed significant differences in gut microbiota composition among the treatment groups (R^2^ = 0.419, *p* = 0.001). These findings suggest that 3'-SL, *B. infantis*, and the synbiotic intervention effectively modulate the composition of the gut microbiota.

Linear discriminant analysis effect size (LEfSe) analysis identified specific bacterial taxa contributing to the differences among groups, spanning from the phylum to genus levels (LDA score > 4.0, *p* < 0.05). For example, g_unclassified_Desulfovibrionaceae, *g_Dubosiella*, and *g_Alistipes* were significantly enriched in the control group, whereas g_unclassified Clostridia_UCG_014 was uniquely abundant in the 3'-SL group. Similarly, *g_Ligilactobacillus* and *g_Bifidobacterium* were predominantly enriched in the *B. infantis* group. In contrast, *g_Allobaculum*, *g_Akkermansia*, and *g_Lactobacillus* exhibited marked enrichment in the synbiotic group (Figure 5(g,h)).

The species distribution bubble map results showed that synbiotic intervention significantly increased the abundance of *Akkermansia*, which may be an intriguing finding (Figure 5(i)).

Overall, these results demonstrate that 3'-SL, *B. infantis*, and their combination positively influence the composition and diversity of the gut microbiota in normal mice, with the synbiotic exerting the most substantial impact, particularly in promoting the abundance of the potential probiotic *Akkermansia*.

#### Effects of 3'-SL, *B.* *infantis*, and the synbiotic on gut microbiota in dss-induced colitis mice.

**Figure 6.**
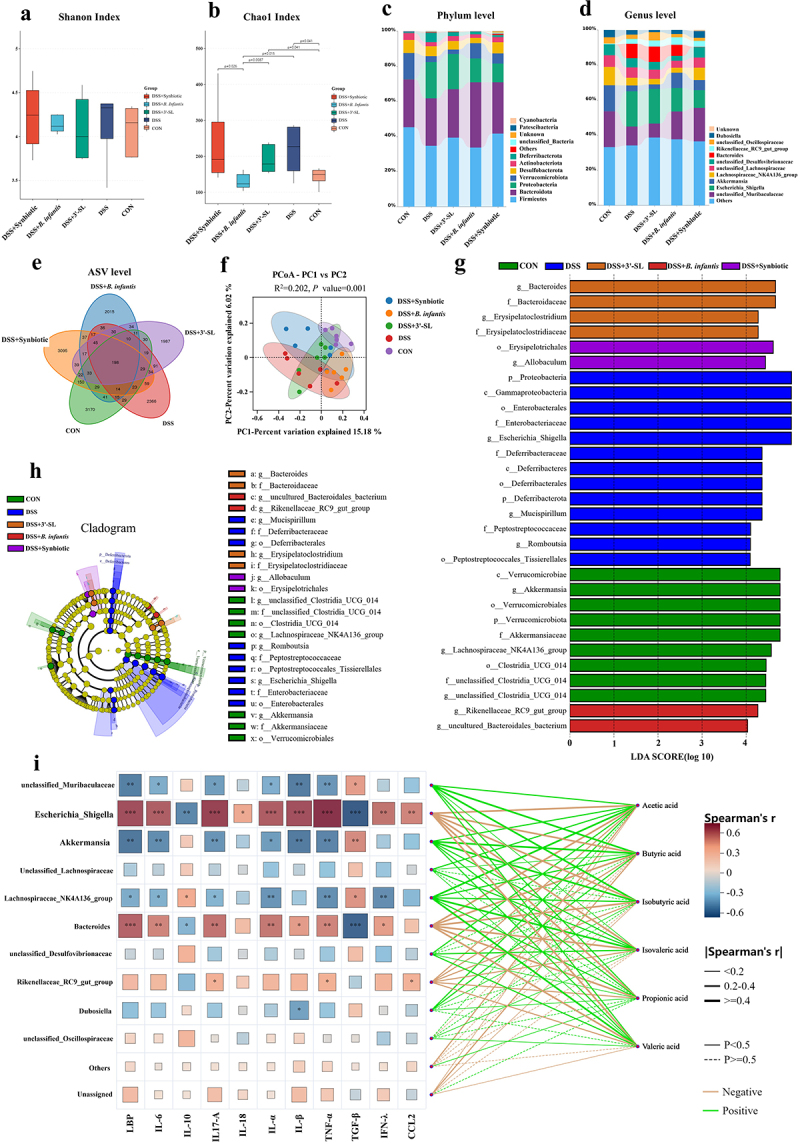
The composition of gut microbiota in DSS-induced mice treated with 3'-SL, *B. infantis*, and the synbiotic (*n* = 6 per group). Mice were administered PBS, DSS, DSS+3'-SL, DSS+*B. infantis*, or DSS+the synbiotic for 7 days, and colonic contents were collected for 16s rRNA sequencing. Alpha diversity is represented by the (a) Chao1 index and (b) Shannon index. (c) Phylum-level composition. (d) Genus-level composition. (e) Venn diagram depicting the common and unique amplicon sequence variants (ASVs) in the five groups. (f) PCoA plots based on the binary Jaccard among the five groups. (g) LEfSe plots showing the differentially abundant microbiota based on ASV level (LDA > 4) across the five groups. (h) Evolutionary branch diagram of LefSe analysis based on classification information. (i) Spearman correlation analysis between colon flora, serum cytokines, and cecum SCFA production. Additional note: some taxa, such as Rikenellaceae RC9 gut group and unclassified Muribaculaceae, were classified at the family level but are displayed in the Genus-level bar plots due to database limitations. These taxa were included in the visualization to maintain consistency across all taxonomic levels.

The gut microbiota composition in DSS-induced colitis mice underwent significant alterations following treatment with 3'-SL, *B. infantis*, and the synbiotic. At both the phylum and genus levels, the treatment groups exhibited distinct microbial profiles. The α-diversity analysis, assessed using the Chao1 and Shannon indices, reflected the richness and diversity of microorganisms at the genus level, as determined by the Wilcoxon rank-sum test (Figures 6(a,b)). While no significant differences were observed in the Shannon index, the Chao1 index revealed significant variations in microbial richness (CON vs. DSS, *p* = 0.041; CON vs. DSS + 3'-SL, *p* = 0.041; DSS vs. DSS+*B. infantis*, *p* = 0.0015; DSS + 3'-SL vs. DSS+*B. infantis*, *p* = 0.0087; DSS+*B. infantis* vs. DSS + synbiotic, *p* = 0.026).

At the phylum level (Figure 6(c)), DSS treatment significantly increased the relative abundances of Proteobacteria and Deferribacterota while decreasing those of Firmicutes, Bacteroidota, Verrucomicrobiota, and Actinobacteriota. Treatments with 3'-SL, *B. infantis*, and the synbiotic maintained the overall abundances of Firmicutes and Bacteroidota compared to DSS-induced mice. Notably, *B. infantis* and the synbiotic effectively suppressed the expansion of Proteobacteria and restored Verrucomicrobiota abundance.

At the taxonomic level analyzed (Figure 6(d)), DSS treatment resulted in an increased abundance of the pathogenic genus *Escherichia-Shigella*, while significantly reducing the levels of beneficial taxa, including unclassified Muribaculaceae, unclassified Oscillospiraceae, *Akkermansia*, *Lachnospiraceae NK4A136 group*, and *Dubosiella*. Treatment with 3'-SL significantly enhanced the abundance of unclassified Oscillospiraceae and *Rikenellaceae RC9 gut group*, but had limited effects on reducing *Escherichia-Shigella* levels. In contrast, *B. infantis* treatment effectively suppressed the abundance of *Escherichia-Shigella* while promoting beneficial bacteria such as unclassified Muribaculaceae, *Akkermansia*, and *Rikenellaceae RC9 gut group*. The synbiotic treatment exhibited the most pronounced effects, significantly enriching the abundance of unclassified Muribaculaceae, unclassified Desulfovibrionaceae, *Akkermansia*, *Lachnospiraceae NK4A136 group*, and *Dubosiella*, while most effectively reducing the abundance of *Bacteroides* and the pathogenic *Escherichia-Shigella.*

Venn diagram analysis revealed that the synbiotic group had a similar ASV count to the control group (3095 vs. 3170), while the 3'-SL and *B. infantis* groups showed comparable ASV counts (1987 vs. 2015) (Figure 6(e)). Principal Coordinates Analysis, based on the binary Jaccard index, demonstrated distinct clustering of gut microbiota structures at the genus level among the treatment groups, indicating notable shifts in microbiota composition (Figure 6(f)). Furthermore, PERMANOVA analysis confirmed significant differences in microbiota composition across the treatment groups (R^2^ = 0.202, *p* = 0.001).

Linear discriminant analysis effect size was employed to identify bacterial taxa that were differentially enriched across the treatment groups, spanning from phylum to genus levels (LDA score > 4.0, *p* < 0.05) (Figures 6(g,h)). g_unclassified Clostridia UCG 014, *g_Akkermansia*, and *g_Lachnospiraceae* NK4A136 group, were enriched in the control group, while *g_Escherichia-Shigella*, *g_Mucispirillum*, and *g_Romboutsia* dominated in the DSS-induced mice. In the 3'-SL group, *g_Bacteroides*, and *g_Erysipelatoclostridium* were enriched, while g_uncultured Bacteroidales bacterium and g_Rikenellaceae RC9 gut group and were dominant in the *B. infantis* group. Notably, only *g_Allobaculum* was enriched in the synbiotic group.

Spearman correlation analysis of colonic microbiota, serum cytokines, and cecal SCFAs production revealed that, during DSS induction (Figure 6(i)), three bacterial taxa in the colon (*Escherichia-Shigella*, *Bacteroides*, and Rikenellaceae RC9 gut group) were negatively correlated with SCFA production in the cecum. In contrast, five key taxa (unclassified Muribaculaceae, unclassified Lachnospiraceae, unclassified Desulfovibrionaceae, *Akkermansia*, *Lachnospiraceae NK4A136 group*, and *Dubosiella*) showed positive correlations with cecal SCFA production. These bacterial taxa, along with changes in SCFA levels, significantly influenced serum cytokine regulation during the development of DSS-induced colitis.

These results indicate that 3'-SL, *B. infantis*, and the synbiotic significantly modulated gut microbiota composition in DSS-induced mice, with the synbiotic exerting the most pronounced effect. Among these treatments, the synbiotic most effectively reshaped microbiota composition at both the phylum and genus levels, particularly by promoting beneficial bacteria such as *Akkermansia* and unclassified Muribaculaceae, while suppressing potentially harmful *Escherichia-Shigella*. This finding suggests that the synbiotic could serve as an effective microbiota-regulating strategy to enhance cecal short-chain fatty acid production and modulate serum cytokine levels, thereby alleviating DSS-induced colitis.

## Discussion

Dietary interventions are increasingly recognized as effective strategies for modulating the gut ecosystem and microbial metabolism, offering promising avenues for colitis prevention.^7^ Among these, sialylated milk oligosaccharides, particularly 3'-SL, have demonstrated significant prebiotic properties, fostering the growth of *Bifidobacterium* and contributing to a healthier intestinal microbiota.^10^ Beyond microbiota modulation, 3'-SL exhibits a broad spectrum of health benefits, including antibacterial and antiviral properties, prevention of necrotizing enterocolitis, immunomodulation, epithelial cell response regulation, cognitive development, and improvements in metabolic health and cardiac function.^9,25,26^

While previous studies have explored the prebiotic potential of 3'-SL and the probiotic effects of *B. infantis* separately, this study introduces a novel approach by combining both, demonstrating enhanced effects compared to each treatment individually. This combination highlights synergistic benefits in reducing intestinal inflammation, restoring barrier function, and modulating gut microbiota balance. Specifically, the combination of 3'-SL and *B. infantis* promotes the growth of beneficial bacteria, such as *Akkermansia* and *Bifidobacterium*, which play a key role in cross-feeding other bacteria to produce SCFAs. These SCFAs, in turn, help strengthen the intestinal barrier and reduce inflammation – an area that has been less explored in previous studies. This combined treatment not only modulates the gut microbiota but also outperforms individual treatments in promoting a healthy microbial ecosystem. Histopathological and immunological analyses further support the therapeutic efficacy of the synbiotic treatment. Together, these findings propose a promising new strategy for the prevention and treatment of ulcerative colitis.

### Synergistic modulation of gut microbiota by 3'-SL and *B.* *infantis*

Previous studies have shown that variations in lacto-oligosaccharide content in human breast milk influence the colonization of intestinal microbes in breastfed infants.^27^ Among these, HMOs play a pivotal role in fostering *Bifidobacterium* interactions within the intestinal ecosystem.^28^ Beyond infancy, supplementation with a synbiotic composed of HMOs and *Bifidobacterium* has been shown to induce reversible engraftment in healthy adult microbiomes, even in the absence of antibiotic intervention. Moreover, targeted modulation of dysbiotic adult microbiomes using a human-milk-derived synbiotic effectively reshapes gut microbial composition and metabolic profiles.^29,30^ This study extends these findings by confirming the association between 3'-SL and *B. infantis*, further highlighting their synergistic role in gut microbiota modulation.

Through 16S rRNA sequencing of fecal samples in mice prior to the DSS challenge, we observed that 3'-SL and *B. infantis* exerted both overlapping and distinct effects on gut microbiota composition. Specifically, the 3'-SL intervention enriched unclassified Clostridia_UCG_014, a taxon potentially involved in carbohydrate metabolism. This suggests that 3'-SL may serve as a selective metabolic substrate for certain beneficial taxa. In contrast, *B. infantis* supplementation primarily supported its own proliferation, reinforcing its well-documented role in colonization of the gut.

Nevertheless, both interventions successfully increased the abundance of beneficial symbiotic bacteria, including *Ligilactobacillus*, which is associated with gut barrier protection. Interestingly, the combined intervention of 3'-SL and *B. infantis* not only leveraged the individual benefits of each component but also exhibited a unique advantage in promoting the growth of *Akkermansia*, a promising probiotic linked to intestinal barrier integrity and IBD management.

These findings underscore a critical distinction between probiotic and synbiotic interventions. While *B. infantis* primarily enhances *Bifidobacterium* colonization, the addition of 3'-SL may create a more favorable gut environment, potentially by enhancing mucosal nutrient availability and supporting cross-feeding interactions. This could explain why the combined intervention uniquely enriched *Akkermansia*, a relatively underexplored but highly beneficial microbe in gut health. Given the role of *Akkermansia* in mucin degradation and SCFA production, its enrichment could represent a novel approach to optimizing microbiota composition and preventing colitis by improving gut barrier function.

### DSS-induced dysbiosis and restores gut homeostasis

Dysbiosis, characterized by a reduction in beneficial symbionts and an overgrowth of harmful pathobionts, is a hallmark of IBD.^31^ Consistent with previous studies, our findings reveal that DSS-induced colitis led to a significant expansion of *Escherichia-Shigella* within the colon, a bacterial group strongly associated with gut inflammation and microbial dysregulation. This overgrowth contributes to the breakdown of microbial homeostasis, exacerbating intestinal imbalance and inflammation, which are key drivers of colitis progression.

While supplementation with 3'-SL alone during DSS-induced colitis effectively enriched beneficial bacteria, including unclassified *Oscillospiraceae* and *Rikenellaceae RC9 gut group*, it had limited efficacy in suppressing *Escherichia-Shigella*. This could be attributed to the metabolic adaptability of pathogenic microbes, particularly their ability to utilize sialic acid residues as a nutrient source. Pathogens such as *Escherichia-Shigella* possess sialidases and other enzymes capable of degrading sialic acid, allowing them to capitalize on 3'-SL-derived substrates to support their expansion. This metabolic advantage enables them to outcompete beneficial bacteria, including *Bifidobacterium* and sialic acid-utilizing species such as *Akkermansia*, thereby reinforcing gut inflammation and worsening dysbiosis. As a result, 3'-SL alone may provide insufficient protection against inflammation-associated pathobionts, limiting its overall therapeutic potential in microbiota restoration.

In contrast, both *B. infantis* and synbiotic treatments successfully preserved *Akkermansia* abundance while concurrently reducing *Escherichia-Shigella* overgrowth. Notably, synbiotic treatment exhibited the strongest inhibitory effect on *Escherichia-Shigella*, underscoring its superior therapeutic potential in modulating gut microbiota and alleviating colitis. This enhanced efficacy may stem from the synergistic effects of *B. infantis* and 3'-SL, where *B. infantis* actively outcompetes pro-inflammatory pathobionts, while 3'-SL fosters a more favorable gut environment that supports beneficial taxa. This synergy appears to enhance microbial resilience and reduce dysbiotic imbalances, making synbiotic therapy a more robust strategy for restoring gut microbial homeostasis.

### Dose-dependent effects of *B.* *infantis* and 3'-SL on colitis and gut homeostasis

This study demonstrated that varying doses of 3'-SL and *B. infantis* exhibited distinct therapeutic effects against DSS-induced colitis in mice. The therapeutic efficacy of *B. infantis* was dose-dependent, with 1 × 10^10^ CFU/day showing the strongest effect. In contrast, 3'-SL did not follow a dose-dependent pattern, with the optimal therapeutic dose being 25 mg/day. This non-dose-dependent effect may indicate diminishing returns or potential adverse effects at higher doses, suggesting that the gut’s ability to process and utilize 3'−SL may be limited once a certain threshold is surpassed.

Mucus sialylation plays a pivotal role in maintaining intestinal host-commensal homeostasis.^32^ Moreover, breast milk oligosaccharides, such as sialyllactose, influence mucosal immunity and bacterial colonization by modifying the balance of sialic acid and fucose in the gut, which in turn alters mucin structure and function. Excessive accumulation of sialic acid or fucose can be utilized by pathogenic bacteria, potentially disrupting the gut environment and compromising intestinal health.

Exposure to sialyllactose during infancy has been shown to impact bacterial colonization in the intestine. Elevated levels of α2,3-linked sialylated structures could prime innate immune cells, thereby increasing susceptibility to colitis.^33,34^ Given that 3'-SL carries sialic acid, excessive accumulation in the gut may disrupt the delicate balance between mucin sialylation and fucosylation – both of which are essential for maintaining gut homeostasis. Therefore, while 3'-SL could offer therapeutic benefits, the strategic introduction of *B. infantis* appears to be a more effective means of maintaining intestinal homeostasis.

In clinical applications, it is vital to carefully consider the dose–response effects of both *B. infantis* and 3'-SL to ensure therapeutic efficacy while avoiding potential imbalances in gut microbial composition. Furthermore, understanding the precise mechanisms of sialylation and microbial interactions in response to these treatments will be critical for optimizing their use in inflammatory bowel disease management.

### Synergistic effects of *B.* *infantis* and 3'-SL on intestinal barrier and inflammation

This study explored the synergistic effects of *B. infantis* and 3'-SL as a synbiotic treatment for DSS-induced colitis. The synbiotic treatment was significantly more effective than either component alone in alleviating colitis symptoms. This enhanced efficacy is likely due to the synergistic interaction between *B. infantis* and 3'-SL in modulating gut microbiota and improving intestinal homeostasis. A similar mechanism has been observed with galactooligosaccharides and *Limosilactobacillus reuteri*, which alleviate inflammation by producing pentadecanoic acid, enhancing tight junction proteins, and suppressing NF-κB signaling.^35^

The intestinal barrier is critical for maintaining gut homeostasis and preventing pathogen invasion.^36^ Compared with *B. infantis* and 3'-SL alone, synbiotic treatment better restored mucosal barrier function by reducing plasma levels of LPS-binding protein (LBP) and upregulating tight junction proteins (ZO-1, occludin, and claudin-1). Additionally, goblet cell-derived mucins, essential for suppressing colitis,^37^ were significantly upregulated following synbiotic intervention. These results highlight the dual action of the synbiotic in fortifying both the physical and functional components of the intestinal barrier.

Pro-inflammatory cytokines, such as IL-6, IFN-α, and IL-1β, exacerbate colitis, while anti-inflammatory cytokines (e.g., IL-10, TGF-β) mitigate intestinal damage.^38^ Supplementation with *Bifidobacterium infantis* EVC001 has been shown to mitigate inflammatory responses by reducing Th2 and Th17 cytokines, enhancing immunoregulatory factors like galectin-1.^39–43^ Our data demonstrated that synbiotic treatment restored IL-10 and TGF-β levels more effectively than individual treatments, highlighting its superior anti-inflammatory effect. Both IL-10 and TGF-β are key cytokines involved in maintaining immune tolerance and regulating excessive inflammation. The restoration of IL-10 and TGF-β levels through synbiotic treatment is especially significant for achieving long-term therapeutic outcomes in colitis. These findings underscore the therapeutic potential of *B. infantis* and 3'−SL as a synbiotic treatment that may offer both immediate relief and sustained immune modulation in colitis management.

### Synbiotic-induced SCFA production and the key role of *Akkermansia*

SCFAs are vital metabolites that maintain intestinal homeostasis and enhance epithelial integrity. Previous studies have shown that combining *Bifidobacterium longum* subsp. *infantis* with human milk oligosaccharides synergistically enhances SCFA production in ex vivo models.^44^ Our results indicate that the synbiotic treatment significantly increased the levels of SCFAs in the cecum of mice, particularly acetic acid and butyric acid, when compared to treatments with *B. infantis* or 3'-SL alone. These SCFAs serve as energy substrates for colonic epithelial cells and suppress inflammation by activating GPR41 and GPR43 signaling pathways, regulating immune responses, and reinforcing tight junction integrity.^45,46^

Cross-feeding interactions between *Bifidobacteria* and butyrate-producing bacteria, such as *Clostridiales* and *Akkermansia*, significantly enhanced SCFA production in the synbiotic group.^47–49^ Among these, *Akkermansia muciniphila* played a pivotal role by degrading mucin into substrates that support the growth of butyrate producers, such as *Faecalibacterium prausnitzii* and *Roseburia*.^50^

Our data demonstrated that *Akkermansia* acted as a critical mediator in the anti-inflammatory effects of synbiotic treatment. Synbiotic treatment maintained *Akkermansia* abundance during DSS-induced colitis. *Akkermansia* is known to degrade mucin into SCFAs, supporting intestinal barrier integrity and modulating immune responses.^51,52^ Its ability to utilize HMOs, structurally similar to mucin, further enhances its role in synbiotic efficacy. For instance, supplementation with 2’-fucosyllactose increased the expression of bacterial glycosyl hydrolases involved in mucin glycan degradation, significantly increasing *Akkermansia* abundance.^53^ The structural attributes of 3'-SL, particularly its terminal sialic acid, may facilitate cross-feeding mechanisms, where one bacterial species produces metabolites that benefit another, promoting SCFA production.^54^

### Synergistic interactions between HMOs, mucin glycosylation, and gut microbiota

HMOs and mucin glycosylation share structural similarities and are essential for shaping gut microbiota composition and maintaining gut barrier integrity. Mucin, a key component of intestinal secretions, is extensively glycosylated, with over 80% of its mass comprised O-glycans.^55^ As a glycoprotein, mucin is heavily glycosylated, providing carbon sources for mucolytic bacteria.^56^ These glycans, which include GalNAc, GlcNAc, fucose, galactose, mannose, and sialic acid, play a critical role in microbial interactions and the balance of the gut microbiota.

Studies have shown that various gut bacteria, particularly those found in breastfed infants, can metabolize mucin glycans.^57^ For instance, *Bifidobacterium infantis*, a key bacterium in early life, is associated with reduced mucin glycan degradation, helping to preserve gut barrier function.^58^ *Bifidobacterium* species, as early gut colonizers, thrive due to their saccharolytic abilities, utilizing glycans through mutualistic cross-feeding or resource-sharing mechanisms. This reflects a cooperative metabolic strategy among *Bifidobacterium* strains.^59^ These species are also crucial in mediating cross-feeding on HMO-derived metabolites.^60,61^ Our results show that 3'-SL promotes the growth of beneficial bacteria, such as *Akkermansia* and *Bifidobacterium*, which play a key role in cross-feeding other bacteria to produce SCFAs.

The metabolic pathways involved in HMO utilization include enzymes with distinct structural folds and substrate specificities, which are upregulated in response to HMOs and during co-culture with *Akkermansia muciniphila*, a well-known mucin degrader.^48^ This study suggests that the terminal sialic acid of 3'-SL may enhance its specificity for *Akkermansia*, thereby promoting its growth and mucin-degrading activity. Building on this, our study advances previous research by exploring the mechanistic interactions between 3'-SL and *B. infantis*, revealing how this combination enhances the microbiota-modulating effects of *Akkermansia*. Specifically, 3'-SL, in synergy with *B. infantis*, promotes the growth of *Akkermansia*, facilitating microbial cross-feeding, influencing SCFA production, strengthening gut barrier integrity through host–microbe interactions, and contributing to immune homeostasis. This novel mechanism, which has been scarcely explored, underscores the therapeutic potential of this synbiotic combination in gut health and inflammatory disease management.

### Limitation of this study and future research directions

This study has several limitations. First, murine models have inherent limitations in modeling human physiology due to species-specific differences in gut microbiota composition and immune system responses. Therefore, further validation through human clinical trials or humanized mouse models is essential. Additionally, this study focused solely on the effects of 3'-SL, leaving the potential influence of fucosylated lactose and other sialylated lactose compounds, particularly in combination with *B. infantis*, on mucin glycosylation modifications yet to be explored. The underlying mechanisms regulating mucin glycosylation and its interactions with gut microbes, especially how sialylated structures like 3'-SL influence mucosal immunity, still require further investigation. Moreover, the role of fecal microbiota transplantation (FMT) was not examined in this study, though it presents a promising avenue for future research. Finally, this study did not fully capture the complexity of gut microbiota interactions, and its findings may not be generalizable to all populations, such as those with chronic IBD or varying dietary habits. This highlights the necessity of further research in more diverse cohorts.

## Conclusion

In summary, our study demonstrates that 3'-SL, *B. infantis*, and the combined symbiotic intervention effectively attenuated DSS-induced colitis by reducing clinical symptoms, alleviating intestinal inflammation, and restoring intestinal barrier integrity and immune homeostasis. The synbiotic treatment showed superior efficacy in modulating the gut microbiota, particularly by enriching SCFA-producing bacteria such as *Akkermansia*, which cross-feed to enhance SCFA production. These changes led to increased levels of protective SCFAs, including butyrate, which activated anti-inflammatory and barrier-enhancing pathways to mitigate colonic damage. Our findings offer novel insights into the role of probiotics and HMOs in alleviating IBD, providing potential strategies for the therapeutic and preventive management of IBD and other inflammatory disorders.

## Supplementary Material

Supplemental material of the revised manuscript2_Mingzhi.docx

## Data Availability

The 16S rRNA sequencing data from mouse feces and colon contents have been deposited in the SRA database (https://www.ncbi.nlm.nih.gov/geo/) under accession number PRJNA1198006. The datasets used and analyzed during the current study would be available from the corresponding author Zeqing Lu on request.
