# Supplementary material for: 3'-Sialyllactose and B. infantis synergistically alleviate gut inflammation and barrier dysfunction by enriching cross-feeding bacteria for short-chain fatty acid biosynthesis
Source: Gut Microbes. 2025 Apr 7;17(1):2486512. doi: 10.1080/19490976.2025.2486512 (PMC11988227; doi:10.1080/19490976.2025.2486512)
Supplement: Supplemental material of the revised manuscript2_Mingzhi.docx [file KGMI_A_2486512_SM7644.docx]

Supplemental Methods

**Supplementary Method 1. Purity analysis of 3'-Sialyllactose by UPLC-MS/MS (Suppl. Fig 1C)**

An ACQUITY UPLC system (Waters, USA) coupled with a Xevo TQ mass spectrometer (Waters, USA) was employed for the determination of target analytes. Chromatographic separation was achieved using an Amino Column (100 mm × 2.1 mm, 1.7 μm), maintained at 40 °C. A 5 μL injection volume was used for each analysis. The mobile phase consisted of water with 10 mmol/L ammonium formate (solvent A) and acetonitrile (solvent B), with a flow rate set to 0.4 mL/min. The gradient program began with 5% solvent A, gradually increasing to 25% solvent A within 2 minutes and holding for 1 minute. The percentage of solvent A was then increased to 90% by 4 minutes, where it was maintained for 2 minutes. Subsequently, the gradient was reversed to 5% solvent A by 6.1 minutes, and the column was equilibrated for an additional 1.9 minutes. The total analysis time was 8 minutes.

Detection and identification of 3'-SL were carried out using a Xevo TQ tandem-quadrupole mass spectrometer (Waters, USA), operating in positive electrospray ionization mode with multiple reaction monitoring. Nitrogen was employed as the drying and nebulizing gas, while argon served as the collision gas. The capillary voltage was set to 3.0 kV, and the cone voltage was optimized within a range of 20 to 40 V for each analyte. The source block and desolvation temperatures were maintained at 150 °C and 550 °C, respectively. The flow rates of the desolvation gas and cone gas were 1000 L/h and 50 L/h, respectively.

Chromatographic separation of 3'-SL under these conditions is depicted in Suppl. Fig 1C, showing the retention time and peak intensity of 3'-SL. The 3'-SL peak was observed at a retention time of 6.25 minutes, with an intensity of 7264, indicating high purity. The quantification of 3'-SL was performed by constructing a standard curve. The purity was calculated using the following formula:

Purity (%) = (Peak Area of 3'-SL / Total Peak Area) × 100.

It should be noted that the chromatogram shows minimal baseline noise and no significant interference from other peaks, further confirming the purity of the 3'-SL sample.

**Supplementary Method 2. Acid production assay of *B. infantis*. (Suppl. Fig 1G)**

Acid production was assessed by monitoring the pH of *B. infantis* cultures. A sterile pH probe was used to measure the pH every 2 hours over a 24-hour period during incubation in TPY medium.

**Supplementary Method 3. Enzyme tolerance assays of *B. infantis*. (Suppl. Fig 1H and 1I)**

Bile salt tolerance: 1% inoculum was cultured in TPY medium for 16 hours. After incubation, fresh culture was plated on TPY agar with different bile salt concentrations (ranging from 1 to 5 g/L) and incubated for 36 hours. CFUs were counted to assess growth.

Trypsin resisitance: 1% inoculum was cultured in TPY medium for 16 hours. Fresh culture was plated on TPY agar with 0.2% trypsin and incubated for 36 hours. CFUs were counted to assess survival.

**Supplementary Method 4. HMOs utilization assays of *B. infantis*. (Suppl. Fig 1J)**

HMOs standards, including LNT, LNnT, 2'-FL, 3'-FL, 3'-SL, and 6'-SL (Glycom A/S, Denmark), were added to autoclaved, carbon-free modified MRS medium using a Millipore needle filter to achieve a final concentration of 20 g/L. The medium was inoculated with 1% (v/v) of the bacterial strain, and each strain was cultured under standardized conditions for 16 hours. Growth was monitored by measuring the optical density at 600 nm (OD600), with three biological replicates conducted for each strain.

Supplemental Figures


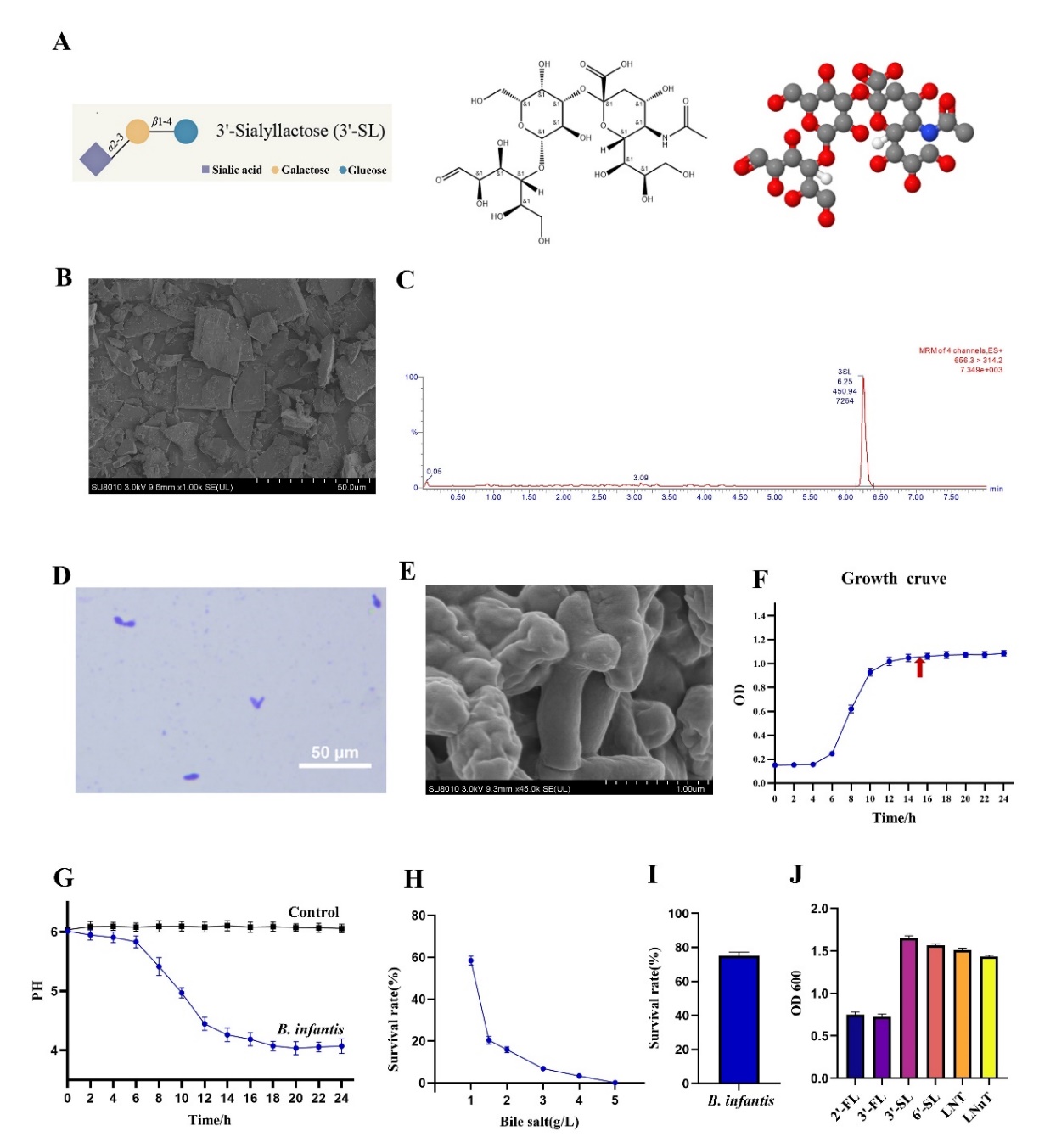


**Supplementary Figure 1. Characterization of 3'-Sialyllactose and *B. infantis*.**

(A) The chemical structures of 3'-Sialyllactose include monosaccharide compositions and linkage types. (B) The SEM images of 3'-Sialyllactose. (C) Purity analysis of 3'-Sialyllactose by UPLC-MS/MS. (D) Gram staining results of *B. infantis*. (E) SEM images *of B. infantis*. (F) Growth curve of *B. infantis* in TPY medium. (G) Determination of acid production capacity of *B. infantis.* (H) Determination of bile salt tolerance of *B. infantis.* (I) Determination of trypsin resisitance of *B. infantis*. (J) HMO utilization capacity of *B. infantis.*


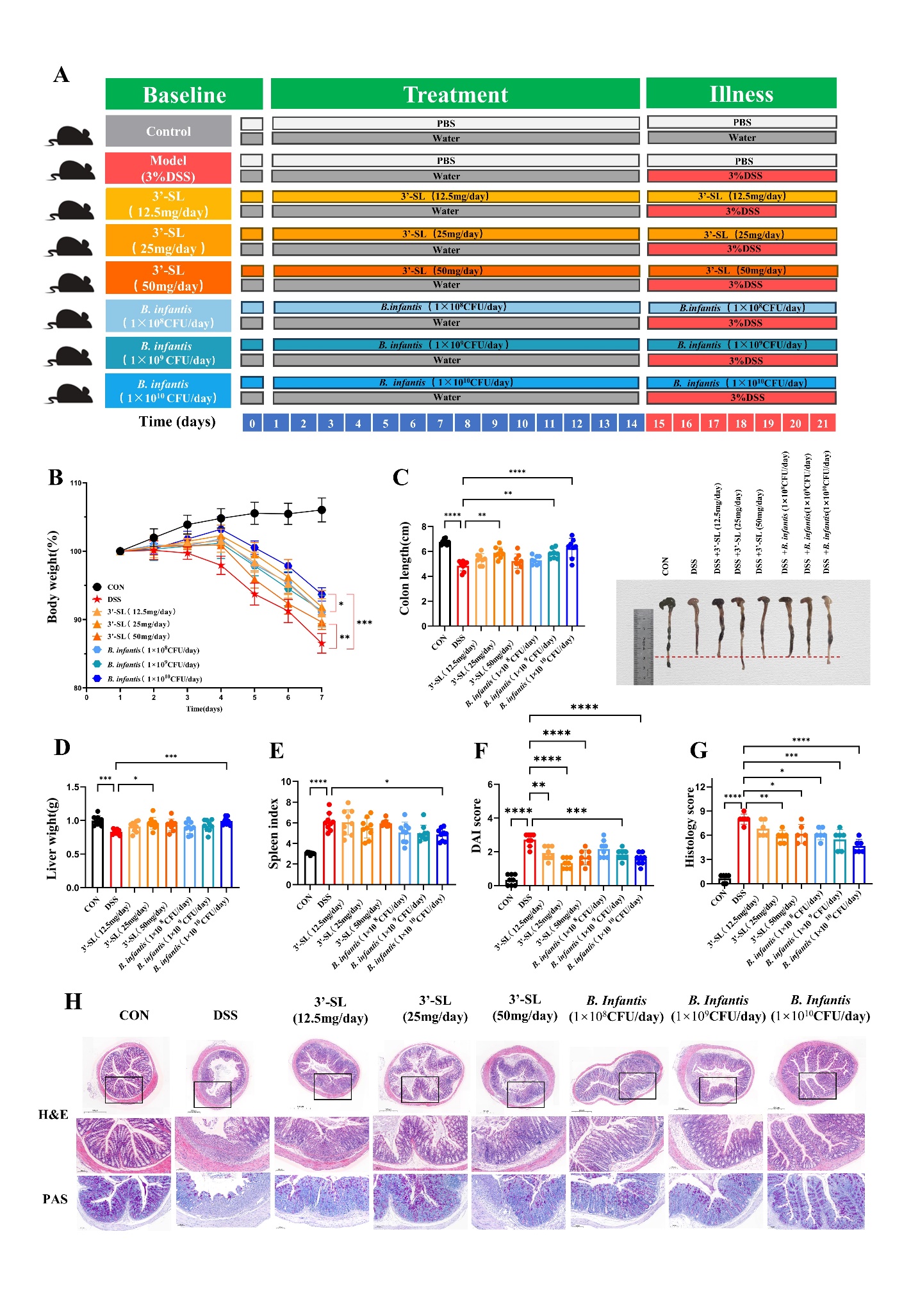


**Supplementary Figure 2. Study on the optimal dose of 3'-SL and *B. infantis* interventions.**

1. Study design. (B) Body weight changes of mice after treatment with different doses and induction of colitis. (C) The colon lengths and macroscopic images of mouse from each group (n = 8 per group). (D) The liver weight of the different mouse groups (n = 8 per group). (E) The spleen index of the different mouse groups (n = 8 per group) (F) The DAI scores of the different mouse groups (n = 8 per group) on the last experimental day. (G) The histology scores of the different mouse groups (n = 6 per group). (H) H&E and PAS staining of colon sections from different groups of mice. Scale bar: 500 μm (upper panel) and 100 μm (lower panel).

Supplemental Tables

**Supplementary Table 1.** **Carbon-free modified MRS liquid medium formulation.**

| **Biochemical reagent component** | **Content** | **Unit** |
| --- | --- | --- |
| Bacterial peptone | 10.0 | g/L |
| Yeast infusion powder | 5.0 | g/L |
| Dipotassium hydrogen phosphate | 2.0 | g/L |
| Diammonium hydrogen citrate | 2.0 | g/L |
| Sodium acetate | 5.0 | g/L |
| Magnesium sulfate | 0.2 | g/L |
| Manganous sulfate | 0.05 | g/L |
| Tween 80 | 1.0 | g/L |
| pH ranges from 6.2 to 6.5 | 25 | ℃ |

**Supplementary Table 2. Primers used for qRT-PCR**

| **Protein** | **Gene** | **Accession number** | **Primer sequences (5'-3')** |
| --- | --- | --- | --- |
| m-GAPDH | *m-GAPDH* | NM_001301503.1 | Forward: 5'-GTTTGGAGGGATCGTGAG-3' |
|  |  |  | Reverse: 5'-TGGGATGGAAACTGTGGAA-3' |
| m-MUC2 | *m-MUC2* | NM_008753.3 | Forward: 5'-AGACGAGCAGAGAGAGCAAG-3' |
|  |  |  | Reverse: 5'-GGCACTTTTACAGACCCAGG-3' |
| m-ZO-1 | *m-ZO-1* | NM_001025336.2 | Forward: 5'-GCAGACGGAAGGAGTCAAG-3' |
|  |  |  | Reverse:5'-AGCTTCTTGAGGAAGGCTTG-3' |
| m-Occludin | *m-Occludin* | NM_001081973.2 | Forward: 5'-AGACCGACGTCACGATGAG-3' |
|  |  |  | Reverse: 5'-CAGGAGGACAGGTAGGAGC-3' |
| m-Claudin1 | *m-Claudin1* | NM_009855.4 | Forward: 5'-AGCCACCATGAGAGAAAGC-3' |
|  |  |  | Reverse: 5'-ACCCACAGCCACCTAGAGT-3' |
| m-GPR43 | *m-GPR43* | NM_009297.4 | Forward: 5'-TGTGAGTCCGAGGTGAGTGG-3' |
|  |  |  | Reverse: 5'-CAGGAACGAGGACATCCACC-3' |
| m-GPR41 | *m-GPR41* | NM_021612.3 | Forward: 5'-ATGGGAGAGGAGGAAGAGGA-3' |
|  |  |  | Reverse: 5'-CTGTAGGTTGTGGAGGAGG-3' |
